# Karyotypes diversity in some Iranian Pamphagidae grasshoppers (Orthoptera, Acridoidea, Pamphagidae): new insights on the evolution of the neo-XY sex chromosomes

**DOI:** 10.3897/compcytogen.v14.i4.53688

**Published:** 2020-11-10

**Authors:** Olesya Buleu, Ilyas Jetybayev, Mohsen Mofidi-Neyestanak, Alexander Bugrov

**Affiliations:** 1 Novosibirsk State University, Pirogova Str. 2, Novosibirsk 630090, Russia; 2 Institute of Systematics and Ecology of Animals, Russian Academy of Sciences, Siberian Branch, Frunze str. 11, 630091, Novosibirsk, Russia; 3 Institute of Cytology and Genetics, Russian Academy of Sciences, Siberian Branch, Pr. Lavrentjeva 10, 630090, Novosibirsk, Russia; 4 Iranian Research Institute of Plant Protection, Hayk Mirzayans Insect Museum, Agricultural Research, Education and Extension Organization, Tehran, Iran

**Keywords:** C-banding, FISH, karyotypes, neo-sex chromosomes, Pamphagidae grasshoppers, ribosomal DNA repeats, telomeric repeat (TTAGG)_n_

## Abstract

For the first time, cytogenetic features of grasshoppers from Iran have been studied. In this paper we conducted a comparative cytogenetic analysis of six species from the family Pamphagidae. The species studied belong to subfamilies Thrinchinae Stål, 1876 (*Eremopeza bicoloripes* (Moritz, 1928), *E. saussurei* (Uvarov, 1918)) and Pamphaginae (*Saxetania paramonovi* (Dirsh, 1927), *Tropidauchen escalerai* Bolívar, 1912, *Tropidauchen* sp., and *Paranothrotes citimus* Mistshenko, 1951). We report information about the chromosome number and morphology, C-banding patterns, and localization of ribosomal DNA clusters and telomeric (TTAGG)_n_ repeats. Among these species, only *S. paramonovi* had an ancestral Pamphagidae karyotype (2n=18+X0♂; FN=19♂). The karyotypes of the remaining species differed from the ancestral karyotypes. The karyotypes of *E. bicoloripes* and *E. saussurei*, despite having the same chromosome number (2n=18+X0♂) had certain biarmed chromosomes (FN=20♂ and FN=34♂ respectively). The karyotypes of *T. escalerai* and *Tropidauchen* sp. consisted of eight pairs of acrocentric autosomes, one submetacentric neo-X chromosome and one acrocentric neo-Y chromosome in males (2n=16+neo-X neo-Y♂). The karyotype of *P. citimus* consisted of seven pairs of acrocentric autosomes, submetacentric the neo-X_1_ and neo-Y and acrocentric the neo-X_2_ chromosomes (2n=14+neo-X_1_ neo-X_2_ neo-Y♂). Comparative analysis of the localization and size of C-positive regions, the position of ribosomal clusters and the telomeric DNA motif in the chromosomes of the species studied, revealed early unknown features of their karyotype evolution. The data obtained has allowed us to hypothesize that the origin and early phase of evolution of the neo-Xneo-Y♂ sex chromosome in the subfamily Pamphaginae, are linked to the Iranian highlands.

## Introduction

Among Pamphagidae grasshoppers, over 300 species inhabit the desert, semidesert and mountainous landscapes of the Palaearctic Region. All of them belong to the subfamilies Thrinchinae and Pamphaginae (Uvarov 1966; Massa 2013; Ünal 2016). Until recently, the Pamphagidae grasshoppers did not attract the attention of cytogenetic researchers. Poor cytogenetic studies of Pamphagidae were associated not only with the low density of their populations, but also with the uniformity of their karyotypes. White (1973) reported a conservative karyotype consisting of 19 acrocentric chromosomes in males and 20 in females with X0♂/XX♀ sex chromosome system. This was confirmed by further studies in Pamphagidae species from Europe, South Africa and China (Hewitt 1979; Camacho et al. 1981; Santos et al. 1983; Cabrero et al. 1985; Fossey 1985; Fu Peng et al. 1989; Mansueto and Vitturi 1989; Vitturi et al. 1993; Warchałowska-Śliwa et al. 1994). Pamphagidae species with the neo-X neo-Y/neo-X neo-X sex chromosome system from Central Asia (Bugrov 1986) has drawn our attention to this family. Cytogenetic information concerning species of *Asiotmethis* Uvarov, 1943 and *Glyphotmethis* Bey-Bienko, 1951 genera (Thrinchinae) and representatives of Nocarodeini tribe (Pamphaginae) from Central Asia, the Caucasus and Transcaucasia, Bulgaria and Turkey have shown variation of sex chromosome systems (Bugrov 1986, 1996; Bugrov and Warchałowska-Śliwa 1997; Bugrov and Grozeva 1998; Bugrov et al. 2016; Jetybayev et al. 2017a). Those variations modified the organization of standard karyotypes, with species showing eight pairs of acrocentric autosomes, one metacentric neo-X chromosome and acrocentric neo-Y chromosome in males (2n♂=18; 16+neo-Xneo-Y) and two metacentric neo-X chromosomes in females (2n♂=18; 16+neo-X neo-X). This karyotype originated from an ancestral Pamphagidae chromosome set, as a result of a Robertsonian translocation of a large acrocentric autosome and acrocentric X chromosome (Bugrov 1986, 1996; Bugrov and Warchałowska-Śliwa 1997; Bugrov and Grozeva 1998; Bugrov et al. 2016).

Moreover, the neo-Y chromosomes found in previously studied Thrinchinae (*Asiotmethis* and *Glyphotmethis* genera) and Pamphaginae (Nocarodeini tribe) species varies in size and content of constitutive heterochromatin. In the karyotypes of some *Glyphotmethis* and *Asiotmethis* species, the neo-Y chromosome is similar in size to its homologous XR-arm of the neo-X chromosome. But unlike the XR-arm of the neo-X chromosome the neo-Y chromosome showed two small interstitial C-bands near the pericentromeric region. In the karyotypes of all Nocarodeini species, the neo-Y chromosome is smaller than the XR-arm of the neo-X chromosome. But unlike the XR-arm of the neo-X chromosome the neo-Y chromosome showed a large pericentromeric C-band and two or three large subproximal interstitial C-bands located close to each other (Bugrov and Grozeva 1998; Bugrov et al. 2016; Jetybayev et al. 2017b). Based on these results it was suggested that neo-Y chromosomes arose independently in two different evolutionary lineages (Thrinchinae and Pamphaginae) and underwent a significant degradation process in Nocarodeini (Jetybayev et al. 2017b). Further evolution of the neo sex chromosomes in the Nocarodeini tribe is associated with the origination of the neo-X_1_X_2_Y♂/neo-X_1_X_1_X_2_X_2_♀ sex chromosome system. Such neo-sex chromosome system was observed in *Paranothrotes opacus* (Brunner von Wattenwyl, 1882) as a result of a Robertsonian translocation of the neo-Y chromosome with an autosome (Bugrov et al. 2016).

Analysis of the geographical distribution of Pamphagidae species with neo-sex chromosomes allowed the assumption that the origin of this type of sex chromosome system may occur in the Western Asian region (Jetybayev et al. 2017a). To test this hypothesis, we acquired data on karyotypes of previously unstudied Pamphagidae species from Iran (Fars, Khorosan-e Razavi and Qazvin provinces) (Table 1). Iran is one of the main centres of species diversity of Pamphagidae grasshoppers. Currently near 110 species from 21 genera of Pamphagidae, belonging to the Thrinchinae and Pamphaginae subfamilies, originate from this area (Mistshenko 1951; Shumakov 1963; Mirzayans 1998; Hodjat 2012; Ünal 2016). The diversity of Iranian Pamphagidae is most significant in the Palearctic Region compared with Europe (52 species), North Africa (101 species), Asia Minor (66 species) and Central Asia (almost 78 species) (Bey-Bienko and Mistshenko 1951; Shumakov 1963; Sergeev 1995; Massa 2013; Ünal 2016).

The present study reports the results of our comparative analysis of the karyotypes, C-banding patterns, distribution of clusters of telomeric (TTAGG)_n_ repeats and ribosomal DNA (rDNA) in the chromosomes of the species studied. We hope that this study will provide the motivation for further cytogenetic study of Iranian grasshoppers.

## Material and methods

### Material collection

Males of the *Eremopeza saussurei* (Uvarov, 1918), *E. bicoloripes* (Moritz, 1928) belonging to the Thrinchinae, and *Saxetania paramonovi* (Dirsh, 1927), *Tropidauchen escalerai* Bolívar, 1912, *Tropidauchen* sp. (Tropidauchenini), *Paranothrotes citimus* Mistshenko, 1951 (Nocarodeini) from the subfamily Pamphaginae were collected in the early summer season (1^st^ to 12^th^ June, 2018) in mountain and semidesert landscapes in Iran (Table 1).

**Table 1.** List of species, collection locations and number of specimens of the studied Pamphagidae species.

Taxa	Species	Location	Number of males
Thrinchinae Thrinchini	*Eremopeza saussurei* (Uvarov, 1918)	Iran, Fars Prov., Zagros Range, 1433 m. asl. 29°25’54.9"N, 052°46’20.0"E	7
	*Eremopeza bicoloripes* (Moritz, 1928)	Iran, Khorosan-e Razavi Prov., 60 km, N. of Mashhad, Ferizi vil. vicinities, ~1800 m. asl.	5
Pamphaginae Nocarodeini	*Paranothrotes citimus* Mistshenko, 1951	Iran, Qazvin Prov., Alborz Range, Qazvin town vicinities, 2380 m. asl. 36°7’29.0"N, 50°40’ 25"E	1
Pamphaginae Tropidauchenini	*Saxetania paramonovi* (Dirsh, 1927)	Iran, Khorosan-e Razavi Prov., 60 km, N. of Mashhad, Ferizi vil. vicinities, ~1800 m. asl.	10
	*Tropidauchen escalerai* Bolívar, 1912	Iran, Fars Prov., Zagros Range, Estahban, Runiz town vicinities, 1800 m. asl.	1
	*Tropidauchen* sp.	Iran, Fars Prov., Zagros Range, 2800 – 3200 m. asl. 30°23’10.1"N, 51°55’35.2"E	7

### Methods

#### Fixation, chromosome preparations, C-banding and fluorescence *in situ* hybridization (FISH)

The 0.1% colchicine solution was injected into the abdomens of collected males. After 1.5–2 hours, their testes were dissected and placed into a 0.9% solution of sodium citrate for 20 minutes. Then the testes were fixed in 3:1 (ethanol : glacial acetic acid) for 15 minutes. Thereafter, fixed testes were stored in 70% ethanol in a refrigerator at 4 °C until used. Air-dried chromosome preparations were made by squashing testis follicles in 45% acetic acid and subsequently freezing them in dry ice.

The constitutive heterochromatin was identified by C-banding, using the technique described by Sumner (1972) with minor modifications. Slides were treated with 0.2 N HCl for 15–20 minutes at room temperature then incubated in a saturated solution of Ba(OH)_2_ at 61°C for three to five minutes, rinsed in tap water and incubated in 2×SSC at 61 °C for 60 minutes. After washing in distilled water, the slides were stained with 2% Giemsa solution on Sorensen’s phosphate buffer for 30 to 60 minutes.

Fluorescence *in situ* hybridization (FISH) with telomeric (TTAGG)_n_ DNA probes and rDNA genes on meiotic chromosomes was carried out according to the protocol by Pinkel (1986) with modifications as described in previous studies (Rubtsov et al. 2000). Telomeric repeats (TTAGG)_n_ were generated by the non-template PCR method with 5’-TAACCTAACCTAACCTAACC-3’ and 5’-TTAGGTTAGGTTAGGTTAGG-3’ primers. Further labelling with Tamra-dUTP (Biosan, Novosibirsk, Russia) was performed in 33 additional cycles of PCR as described previously (Sahara et al. 1999). The ribosomal DNA probe was obtained as previously described (Buleu et al. 2017; Jetybayev et al. 2017a). An unlabelled rDNA probe was generated by the polymerase chain reaction (PCR) according to Jetybayev (2017a). The fragments of the 18S rDNA and 28S rDNA genes were labelled in additional PCR cycles with Fluorescein-12-dUTP (Biosan, Novosibirsk, Russia) and mixed together into a single rDNA probe. For the description of karyotype structure, location and size of C-positive regions in chromosomes, the nomenclature previously proposed for Pamphagidae grasshoppers was used (King and John 1980; Santos et al. 1983; Cabrero and Camacho 1986). According to this nomenclature, autosomes were numbered in order of decreasing size (1–9) and classified into three size groups: L – large, M – medium and S – small. The neo-sex chromosomes were named after White (1940). The arms of the neo-X chromosome were referred to as XL and XR. The XL-arm corresponds to the original acrocentric X chromosome and the XR-arm to the translocated acrocentric autosome. The other non-translocated autosome, homologous to the XR-arm, remains acrocentric and is the neo-Y chromosome (White 1940; Hewitt 1979). In the multiple X_1_X_2_Y/X_1_X_1_X_2_X_2_ sex chromosome system, the neo-X_1_ is formed by the XL- and the XR-arms of the neo-X chromosome described above. The neo-Y is biarmed. The short YL-arm corresponds to the neo-Y chromosome described above and the long YR-arm is formed by a second Robertsonian translocation with a second acrocentric autosome. The homologous of non-translocated autosome from this second pair is referred to as the neo-X_2_ chromosome (White 1940; Hewitt 1979).

Microscopic analysis was performed at the Centre for Microscopy of Biological Objects of SB RAS (Novosibirsk, Russia). Chromosomes were studied with an AxioImager M1 (Zeiss, Germany) fluorescence microscope equipped with filter sets #49, #46HE, #43HE and a ProgRes MF (MetaSystems GmbH, Germany) CCD camera. The ISIS5 software package was used for image capture and analysis.

## Results

### *Eremopeza bicoloripes* (Moritz, 1928)

**Figure 1.**
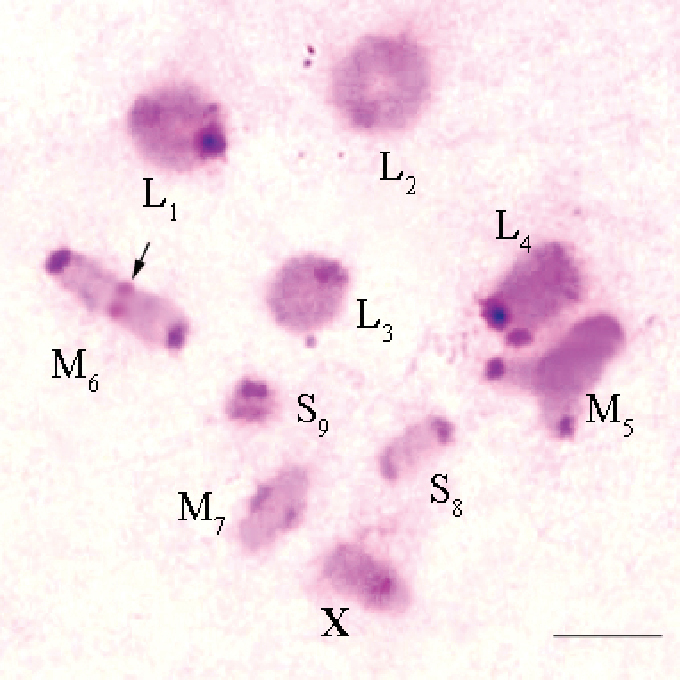
C-banded metaphase I of *Eremopeza bicoloripes*. Arrow indicate the telomeric C-bands in M_6_ autosome bivalent. Scale bar: 10 µm.

The karyotype of *E. bicoloripes* consisted of nine pairs of acrocentric autosomes and one subacrocentric X chromosome in males (2n♂=19; 18AA+X) (Fig. 1). The male meiotic karyotype was represented by four large (L_1_–L_4_), three medium (M_5_–M_7_) and two small (S_8_–S_9_) autosome bivalents and a medium-sized X univalent (Fig. 1).

Distinct pericentromeric C-bands were revealed in all chromosomes of the complement (Fig. 1). Telomeric C-bands were localized in the M_6_ autosome bivalent (Fig. 1).

**Figure 2.**
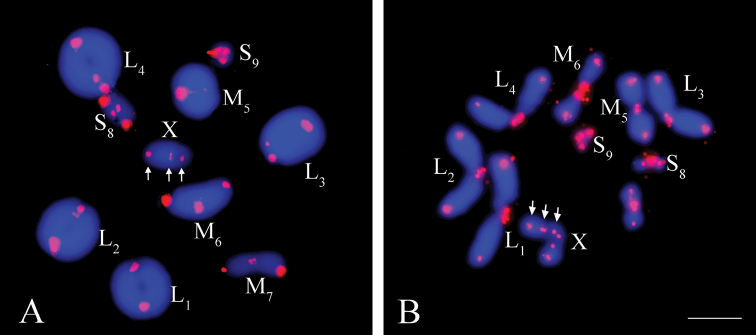
FISH with telomeric (TTAGG)_n_ probe (red) in male meiotic chromosomes of *Eremopeza bicoloripes***A** metaphase I **B** metaphase II. Chromosomes were counterstained with DAPI (blue). Arrows indicate the telomere hybridization signals at the terminal and pericentromeric regions of X chromosome

Telomeric DNA repeats were hybridized at the terminal region of all chromosomes (Fig. 2). In the subacrocentric X chromosome, additional clusters of telomeric DNA were observed in the distal area of the pericentromeric region of the long arm (Fig. 2B).

**Figure 3.**
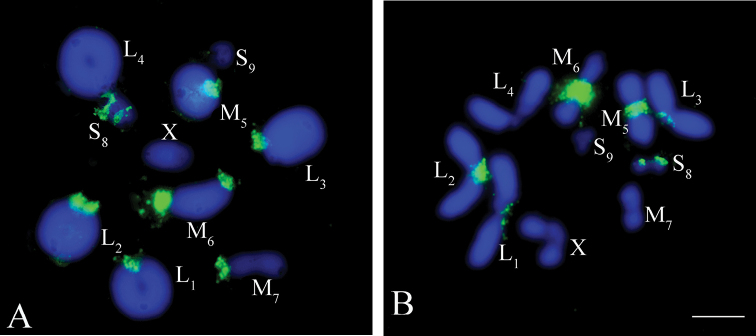
FISH with rDNA genes (green) in male meiotic chromosomes of *Eremopeza bicoloripes***A** metaphase I **B** metaphase II. Chromosomes were counterstained with DAPI (blue). Scale bar: 10 µm.

The clusters of rDNA genes were located at pericentromeric regions in most autosome bivalents, except the L_4_ and S_9_ autosome bivalents and the X chromosome (Fig. 3). In the L_3_ and the M_7_ bivalents, the clusters of ribosomal DNAs were only detected in one of the homologues (Fig. 3A).

### *Eremopeza saussurei* (Uvarov, 1918)

**Figure 4.**
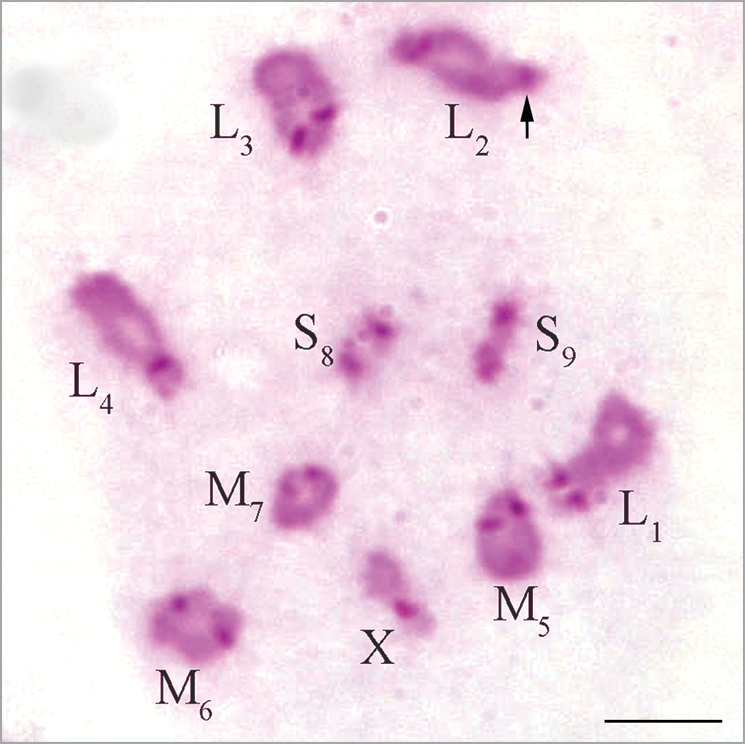
C-banded diakinesis of *Eremopeza saussurei*. Arrow indicates the telomeric C-band in L_2_ autosome bivalent. Scale bar: 10 µm.

The karyotype of *E. saussurei* consisted of nine pairs of autosomes and the X chromosome in males (2n♂=19; 18AA+X). Four autosome bivalents were large (L_1_–L_4_), three were medium (M_5_–M_7_) and two were small (S_8_–S_9_). The X chromosome was of medium-sized. All large autosome bivalents, the M_5_, the M_7_, and the X chromosome were subacrocentric. The M_6_ autosome bivalent was submetacentric. Small (S_8_–S_9_) autosome bivalents were acrocentric (Fig. 4).

Distinct pericentromeric C-bands were revealed in all chromosomes of the complement (Fig. 4). Telomeric C-bands were revealed in long arm of the L_2_ autosome bivalent (Fig. 4).

**Figure 5.**
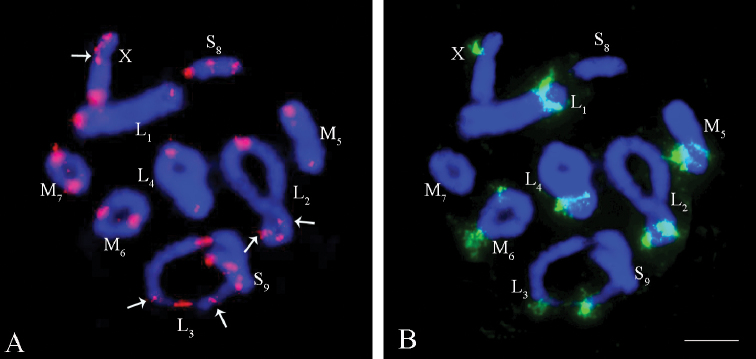
FISH with telomeric (TTAGG)_n_ probe (red) (**A**) and rDNA genes (green) (**B)** in cell at diakinesis of *Eremopeza saussurei*. Arrows indicate the telomere hybridization signals at pericentromeric regions of L_2_ and L_3_ bivalents and X chromosome. Chromosomes were counterstained with DAPI (blue). Scale bar: 10 µm.

Telomeric DNA repeats were observed at terminal regions of all chromosomes (Fig. 5A). Telomeric hybridization signals were also found at pericentromeric regions of two large (L_2_, L_3_) bivalents and the X chromosome (Fig. 5A). Clusters of rDNA genes were observed at pericentromeric regions of six autosome bivalents (L_1_–L_4_, M_5_–M_6_) and X univalent (Fig. 5B).

### *Saxetania paramonovi* (Dirsh, 1927)

**Figure 6.**
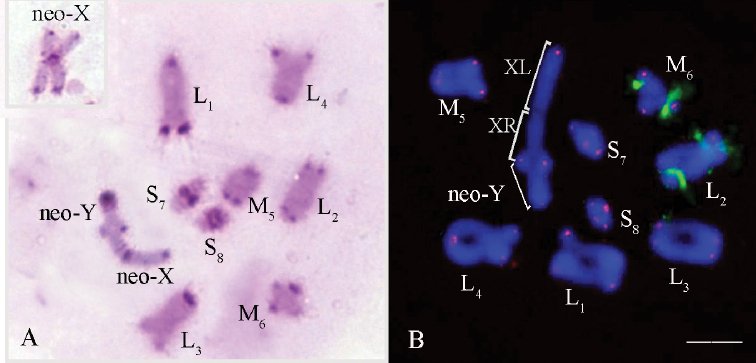
*Saxetania paramonovi***A** C-banded metaphase I **B**FISH with telomeric (TTAGG)_n_ probe (red) and rDNA genes (green) in cell at diakinesis. Chromosomes were counterstained with DAPI (blue) (**B**). Scale bar: 10 µm.

The karyotype of *S. paramonovi* consisted of nine pairs of acrocentric autosomes and an acrocentric X chromosome in males (2n♂=19; 18AA+X). The male meiotic karyotype was represented by four large (L_1_–L_4_), three medium (M_5_–M_7_) and two small (S_8_–S_9_) autosome bivalents. The X chromosome was of medium-sized (Fig. 6A).

Pericentromeric C-bands were revealed in all autosome bivalents and the X chromosome (Fig. 6A). The pericentromeric C-band on one of the homologues in the L_1_ bivalent was noticeably larger than in the other homologue (Fig. 6A). Telomeric C-bands were observed in the M_7_, S_8_ and S_9_ autosome bivalents (Fig. 6A).

Telomeric DNA repeats were only observed in the terminal regions of all chromosomes (Fig. 6B). The clusters of rDNA genes were detected in the pericentromeric region of the L_2_ and L_4_ autosome bivalents and in the proximal interstitial region of the L_3_ autosome bivalent (Fig. 6B).

### *Tropidauchen escalerai* Bolívar, 1912

**Figure 7.**
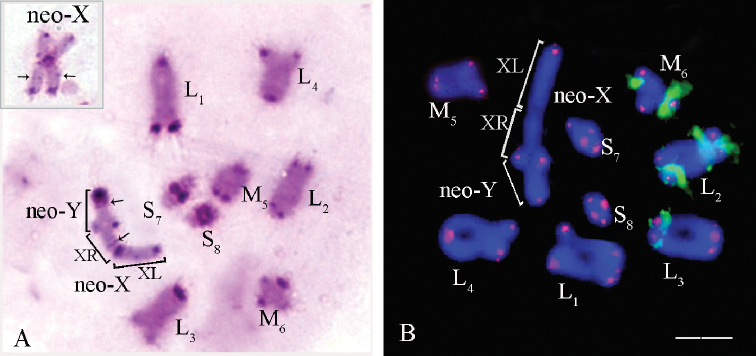
*Tropidauchen escalerai***A** C-banded metaphase I. The inset in the right upper corner shows the neo-X chromosome in meiotic metaphase II. Arrows indicate the C-bands in the XR-arm of the neo-X and neo-Y chromosomes **B**FISH with telomeric (TTAGG)_n_ probe (red) and rDNA genes (green) in cell at metaphase I. Chromosomes were counterstained with DAPI (blue) (**B**). Scale bar: 10 µm.

The karyotype of *T. escalerai* consisted of 18 acrocentric chromosomes (2n=16+neo-Xneo-Y♂): four large (L_1_–L_4_), two medium (M_5_, M_6_) and two small sized (S_7_, S_8_) autosome bivalents (Fig. 7). The neo-X chromosome was metacentric (Fig. 7A, inset). The neo-Y chromosome was acrocentric. During meiosis, the XR-arm of the neo-X and neo-Y usually forms one chiasma at interstitial or subterminal positions (Fig. 7).

Distinct pericentromeric C-bands were found in all autosome bivalents and in the neo-X chromosome (Fig. 7A). The pericentromeric region of the neo-Y chromosome showed a large C-block (Fig. 7A). Tiny interstitial C-bands were observed in the proximal positions in the XR-arm of the neo-X and in the neo-Y chromosomes (Fig. 7A). Telomeric C-bands were detected in the L_1_, L_2_ and L_4_ autosome bivalents and in both arms of the neo-X chromosome (Fig. 7A).

Telomeric DNA repeats were located only at terminal regions of all chromosomes (Fig. 7B). Clusters of rDNA genes were observed in three autosome bivalents (Fig. 7B). Two clusters of rDNA genes were observed in the L_2_ autosome bivalent: the first one located in the proximal interstitial region and the second one in the distal interstitial region (Fig. 7B). In the L_3_ bivalent, the rDNA cluster was localized in the distal area of the pericentromeric region. In the M_6_ bivalent, the cluster of rDNA genes was observed in the interstitial position (Fig. 7B).

### *Tropidauchen* sp.

**Figure 8.**
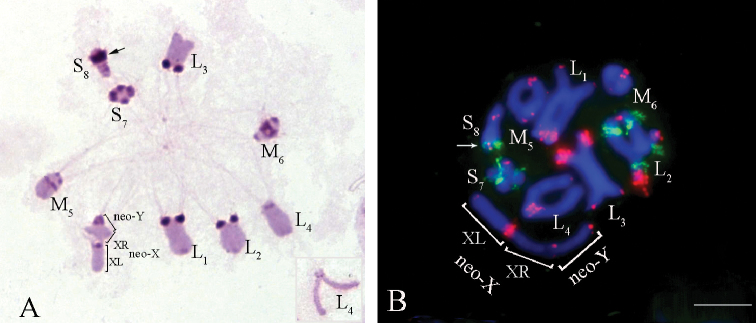
*Tropidauchen* sp. **A** C-banded metaphase I. The inset in the lower left corner shows the L_4_ chromosome in meiotic metaphase II. Arrows indicate the interstitial C-band in the S_8_ autosome bivalent; **B**FISH with rDNA (green) and telomeric (TTAGG)_n_ (red) probes in cell at metaphase I. Arrows indicate the cluster of rDNA genes in the S_8_ autosome bivalent. Chromosomes were counterstained with DAPI (blue) (**B**). Scale bar: 10 µm.

The karyotype of the *Tropidauchen* sp. consisted of 18 chromosomes (2n=16+neo-Xneo-Y♂): three large (L_1_, L_2,_ L_3_), two medium (M_5_, M_6_) and two small (S_7_, S_8_) acrocentric autosome bivalents (Fig. 8). The L_4_ autosome bivalent was subacrocentric (Fig. 8A, inset). The neo-X chromosome was metacentric (Fig. 8A). The neo-Y chromosome was acrocentric. During meiosis, the XR-arm of the neo-X and neo-Y usually forms one chiasma at interstitial or subterminal positions (Fig. 8).

Pericentromeric C-bands were detected in all chromosomes (Fig. 8A). Interstitial C-bands were identified in the M_5_ and S_8_ autosome bivalents (Fig. 8A). In the S_8_ autosome bivalent, one of the homologues had a huge interstitial C-band. The other homologue a thin C-band in the same position (Fig. 8A). Telomeric C-positive block was revealed in the M_6_ and S_7_ autosome bivalents (Fig. 8A).

Telomeric DNA repeats in *Tropidauchen* sp. were localized only at terminal regions of the all autosomes (Fig. 8B). Additional clusters of telomeric repeats were observed in the pericentromeric region of the neo-X chromosome (Fig. 8B). The clusters of rDNA genes were localized in the L_2_, S_7_, and S_8_ autosome bivalent (Fig. 8B). Two clusters of rDNA repeats were observed in the L_2_ bivalent: the first one located in the proximal interstitial region and the second one in the distal interstitial region (Fig. 8B). In the S_7_ autosome bivalent, the cluster of rDNA repeats was revealed at the interstitial region (Fig. 8B). In the S_8_ autosome bivalent, the clusters of rDNA genes were detected only in one homologue (Fig. 8B). This cluster was localized in the proximal position on the border of the C-positive huge band and C-negative chromatin (Fig. 8A, B).

### *Paranothrotes citimus* Mistshenko, 1951

**Figure 9.**
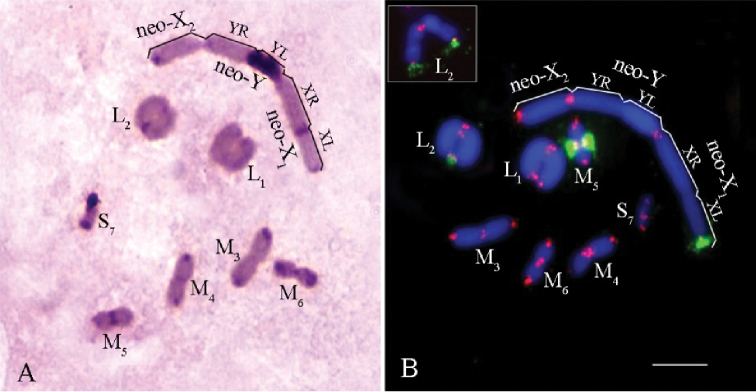
*Paranothrotes citimus***A** C-banded chromosome in metaphase I **B**FISH with rDNA (green) and telomeric (TTAGG)_n_ (red) probes in cell at metaphase I. The inset in the right upper corner shows the L_2_ chromosome in meiotic metaphase II. Chromosomes were counterstained with DAPI (blue) (**B**). Scale bar: 10 µm.

The karyotype of *P. citimus* consisted of 14 autosomes and three neo-sex chromosomes (2n=14+neo-X_1_neo-X_2_neo-Y♂). The karyotype structure was represented by two large (L_1_–L_2_), four medium (M_3_–M_6_) and one small (S_7_) acrocentric autosome bivalents and three neo-sex chromosomes (Fig. 9A). The neo-X_1_ and the neo-Y chromosomes were submetacentric. The neo-X_2_ chromosome was acrocentric. During prophase I of male meiosis the sex chromosomes formed a trivalent consisting of the neo-X_1_, neo-X_2_ chromosomes and the neo-Y chromosome (Fig. 9A).

Distinct pericentromeric C-positive blocks were observed in all chromosomes. The YL-arm of the neo-Y chromosome was completely C-positive (Fig. 9A).

FISH signals of telomeric DNA probe were observed in the terminal regions of all chromosomes (Fig. 9B). The clusters of rDNA genes were localized near the pericentromeric region of the M_5_ and at distal position of L_2_ autosomal bivalents, and at terminal region in the X_1_L-arm of the neo-X_1_ chromosome (Fig. 9B).

The chromosome number, morphology, sex chromosome system, distribution of heterochromatin (C-bands) and location of rDNA and tDNA genes in the studied Pamphagidae species presented in Table 2.

**Table 2.** The chromosome number, chromosomal morphology, sex chromosome system, distribution of constitutive heterochromatin (C-bands) and location of rDNA and tDNA genes in the studied Pamphagidae species.

**Taxa**	**2n♂; FN**	**SD**	**KS**	**CM**	**C-bands**	**rDNA**	**tDNA**
Thrinchinae, Thrinchini							
*Eremopeza bicoloripes*	19; 20	X0	L_1_–L_4_, M_5_-M_7_, S_8_–S_9_, X	all a	p all;	p1,2,3*,4,5,	d all
		XX		X sm	t 1,4,	6,7*,8	dpd X
					5,6,9		
*Eremopeza saussurei*	19; 34	X0	L_1_–L_4_, M_5_–M_7_, S_8_–S_9,_ X	1-4,5,7, X sa;	p all;	p1-4,5,6,7,X	d all
		XX		6 sm;	t 2		dpd 2,3,X
				8,9 a			
Pamphaginae, Nocarodeini							
*Paranothrotes citimus*	14;18	neo-X_1_X_2_Y	L_1_–L_2_, M_3_–M_6_ S_7_	all a	p all;	p5; d2; X_1_L	d all
		neo-X_1_X_1_X_2_X_2_	neo-X_1_	neo-X_1_sm	t X_1_L		
			neo-X_2_	neo-X_2_ a			
			neo-Y	neo-Y sm			
Pamphaginae, Tropidauchenini							
*Saxetania paramonovi*	19;19	X0	L_1_–L_4_, M_5_-M_7_, S_8_–S_9_ X	all a	p all;	p2,4;i3	d all
		XX			t 7,9,8		
*Tropidauchen escalerai*	18;19	neo-XY	L_1_–L_4_, M_5_,M_6_ S_7_, S_8_	all a	p all;	p2i2; p3;i6	d all
		neo-XX	neo-X	neo-X m	i XR, neo-Y;		
			neo-Y	neo-Y a	t 1,2,4, neo-X		
*Tropidauchen* sp.	18;19	neo-XY	L_1_–L_4_, M_5_,M_6_ S_7_, S_8_	1-3,5,6,7,8 a,	p all;	ip2, id2; i7; 8*	d all
		neo-XX	neo-X	4 sa	i 5,8;		dpd neo-X
			neo-Y	neo-X sm neo-Y a	t 6,7		

FN=fundamental number of chromosome arms; SD=sex chromosome system; KS=karyotype structure; L=large; M=medium; S=small; CM=morphology of chromosomes; a=acrocentric; sa=subacrocentric; sm=submetacentric; p=pericentromeric, i=interstitial, t=telomeric; rDNA=clusters of ribosomal DNA; tDNA=telomeric DNA repeats; d=distal; *=in one of the homologues; XR=XR-arm neo-X chromosome; X_1_L=X_1_L-arm of the neo-X_1_ chromosome.

## Discussion

A comparative cytogenetic analysis of Iranian Pamphagidae provides new information about the karyotype evolution in this group of grasshoppers. Two species from the *Eremopeza* Saussure, 1888 genus (Thrinchinae) have the fundamental chromosome number of the Pamphagidae karyotype (2n=19♂). However, unlike the standard Pamphagidae karyotype, in which all chromosomes are acrocentric, in *Eremopeza* subacrocentric chromosomes were found. Early, biarmed chromosomes were found in *Eremopeza festiva* (Saussure, 1888) from Armenia (Bugrov et al. 2016). Two possible paths of the origin of biarmed chromosomes in *Eremopeza* genus may suggested: a) amplification of repetitive elements; b) pericentric inversion. It was shown that in *E. festiva* the presence of all biarmed chromosomes (FN=38) was associated with invasion and amplification of rDNA repeats (Bugrov et al. 2016). In species of *Eremopeza* analyzed in this article not all chromosomes in the sets are biarmed. In *E. bicoloripes*, the X is the only biarmed chromosome and has no clusters of rDNA genes. In *E. saussurei*, most chromosomes in the karyotype have small second arms. The rDNA clusters in this species are located only in pericentromeric regions on biarmed chromosomes, while small arms were not enriched by the rDNA repeats. These observations indicate that the formation of the second arms in *E. bicoloripes* and *E. saussurei* are not associated with the amplification of rDNA repeats. Also, the presence of interstitial telomeric sites in pericentromeric region of some biarmed chromosomes is a strong argument in favor of the inversion hypothesis.

The discovery of some Pamphagidae species with neo-sex chromosome systems supports our hypothesis that the origin of this unusual sex chromosome system is the West Asian region (Jetybayev et al. 2017a). The two species with the neo-sex chromosomes belong to the Tropidauchenini tribe. Previously, the karyotype of only one species, *Saxetania cultricollis* (Saussure, 1887), from this tribe was described. In this species a neo-XY sex chromosome system was found (Bugrov and Warchałowska-Śliwa 1997). Thus, in the Tropidauchenini tribe both the X0 (*S. paramonovi*) and neo-XY sex chromosome systems (*S. cultricollis*, *T. escalerai* and *Tropidauchen* sp.) exist (Figs 6–8). It should be noted that in *S. cultricollis* and *Tropidauchen*, the neo-Y chromosome is very similar to the XR-arm of the neo-X chromosome. During meiosis, these homologous chromosomes form a sex bivalent with one or two chiasmata. The localization of the C-positive regions in the neo-Y chromosome in these species, also does not differ from its homologue, namely the XR-arm of the neo-X. These features indicate that in Tropidauchenini we found the initial stage of neo-XY sex chromosome evolution in the Pamphaginae subfamily. All early studied species of the Nocarodeini tribe (Pamphaginae) possessed a neo-sex chromosome system. In these works, it was emphasized that in Nocarodeini tribe the neo-Y is significantly shorter than the XR and shows a significantly larger heterochromatic region. In the meiosis prophase I, the XR and the neo-Y chromosome of the Nocarodeini species were associated only with the distal region. These features indicate that the Nocarodeini tribe demonstrate the advanced stage of the neo-Y chromosome evolution in Pamphaginae (Bugrov and Grozeva 1998; Bugrov et al. 2016; Jetybayev et al. 2017a, b).

**Figure 10.**
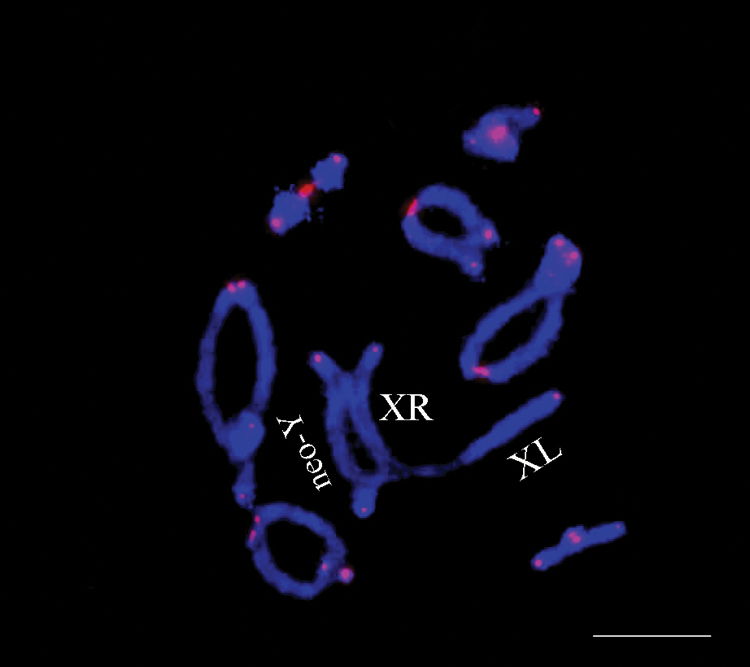
*Saxetania cultricollis*: FISH with telomeric repeats (red) in cells at diakinesis. Chromosomes were counterstained with DAPI (blue). Scale bar: 10 µm.

The fluorescence *in situ* hybridization (FISH) with telomeric probe and rDNA genes is a very useful tool for comparative analysis of karyotype in Orthoptera insects (Warchałowska-Śliwa et al. 2020). In addition, the determination of the position of telomeric and rDNA repeats in chromosomes of many groups of insects made it possible to identify the mechanisms of structural rearrangements (Kuznetsova et al. 2019). It is known that telomeres play an important role in the stability of the eukaryotic karyotype. Basically, telomeric repeats are located at the physical ends of chromosomes in the form of tandem arrays that protect the ends of the chromosomes from attack by exonucleases, degradation and prevent chromosome fusion (Bolzán 2017; Kuznetsova et al. 2019). In chromosome rearrangements the clusters of telomeric repeats may be transferred to interstitial chromosome locations so-called interstitial telomeric sequences (ITSs). Therefore, ITSs may constitute good markers of the occurrence of chromosome rearrangements. We expected to observe ITS in the pericentromeric regions of the neo-X chromosomes in the *Tropidauchenini* tribe. However, the telomeric motif in the Robertsonian translocation site between the X chromosome and the autosome in *T. escalerai* was not observed. Similar results were previously shown in the vast majority of species belonging to the Nocarodeini tribe (Pamphaginae) (Jetybayev et al. 2017a). Additionally, we performed FISH of the telomeric (TTAGG)_n_ probe in chromosomes of the *Saxetania cultricollis* from Turkmenistan. It was also discovered that in *S. cultricollis*, there was no telomeric repeats in the pericentromeric region of the neo-X chromosome (Fig. 10). The absence of telomeric repeats in the pericentromeric region of the neo-X chromosome of these species may indicate that the Robertsonian translocation of the X chromosome and the autosome was accompanied by the deletion of a chromosome fragment containing telomeric DNA repeats. Nevertheless, in *Tropidauchen* sp. we observed telomeric repeats in the pericentromeric region of the neo-X chromosome (Fig. 8B). Previously, the presence of these repeats in the pericentromeric region of the neo-X chromosomes was detected in two species of the *Paranocarodes* Bolívar, 1916 genera (Jetybayev et al. 2017a). It is hardly possible, that in the aforementioned *Paranocarodes* species and *Tropidauchen* sp. the origin of the neo-XY sex system, was different from that of other XY species of Pamphaginae. We suggest that the ITS in these species could occur after pericentric inversion in the neo-X chromosome.

The distribution of rDNA clusters in the chromosomes of the *Saxetania* and *Tropidauchen* species was similar to the distribution of rDNA in the chromosomes of previously studied Pamphaginae species (Vitturi et al. 2008; Bugrov et al. 2016; Jetybayev et al. 2017a). The rDNA clusters were localized on two or three autosome bivalents at the pericentromeric and interstitial regions. One large pair of autosomes carried two rDNA clusters at interstitial position in the proximal and distal regions. Multiple rDNA sites on a single chromosome is a very rare type of rDNA cluster distribution among Acridoid grasshoppers (Cabrero and Camacho 2008; Jetybayev et al. 2012; Palacios-Gimenez et al. 2013). This feature has only been detected in species of the family Pamphagidae (Vitturi et al. 2008; Bugrov et al. 2016; Jetybayev et al. 2017a; Buleu et al. 2019). Our results thus confirm a special type of rDNA cluster localization in the Pamphagidae grasshoppers.

The neo sex chromosome systems were observed in two subfamilies (Thrinchinae and Pamphaginae) of the Pamphagidae grasshoppers. Based on the analysis of the chromosome features (karyotype, C-banding, telomeric (TTAGG)_n_ and rDNA genes) we see that the neo-sex chromosome system in the genera *Saxetania* and *Tropidauchen* in the subfamily Pamphaginae is at a similar level of chromosome evolution to the neo-sex chromosomes in the genera *Glyphotmethis* and *Asiotmethis* of the subfamily Thrinchinae (Bugrov 1996; Jetybayev et al. 2017a). However, the neo-XY system was observed only in several species of the genera *Asiotmethis* and *Glyphotmethis* and no advanced stages of the neo-Y differentiation were observed in this subfamily. Conversely, in the subfamily Pamphaginae, the neo-Y chromosome was observed at different stages of its evolution from the chromosome that is homologous to the autosome (in the tribe Tropidauchenini) to the small heteromorphic mostly heterochromatic (in the tribe Nocarodeini). Furthermore, in the tribe Nocarodeini, we observed an additional stage of the structural evolution of the neo-sex chromosomes: formation of the multiple neo-X_1_X_2_Y♂ sex chromosome system. Previously, this kind of sex chromosome system was identified in the *Paranothrotes opacus* from Armenia (Bugrov et al. 2016). In this paper, we report on a second species with the same type of neo-sex chromosome system and other cytogenetic characters – *Paranothrotes citimus*. It is possible that the evolutionary divergence of the species in the genus *Paranothrotes* could occur on the basis of the neo-X_1_X_2_Y♂sex chromosome system.

**Figure 11.**
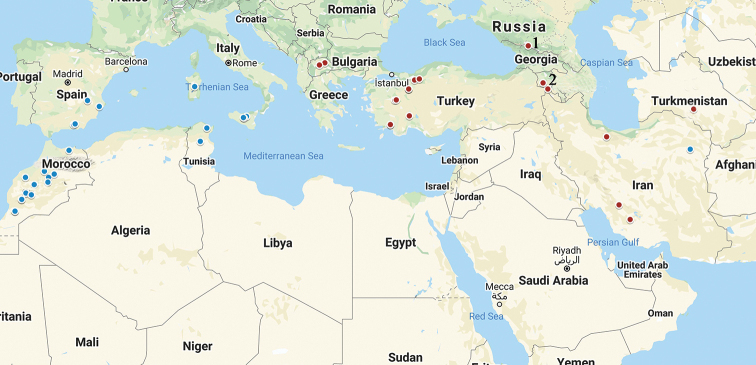
Distribution of Pamphaginae grasshoppers with the X0 (blue circles) and neo-sex chromosomes (red circles). 1 – North Caucasus (Russia). 2 – Armenia.

Analysis of the geographic distribution of Pamphaginae grasshoppers with different types of the sex chromosome systems (Alicata et al. 1976; Camacho et al. 1981; Cabrero et al. 1985; Vitturi et al. 1993; Warchałowska-Śliwa et al. 1994; Bugrov 1996; Bugrov and Warchałowska-Śliwa 1997; Bugrov and Grozeva 1998; Bugrov et al. 2016; Jetybayev et al. 2017a; Buleu et al. 2019) confirmed that species with the neo-sex chromosomes widespread mainly in Western Asia (Fig. 11). The finding of species with the sex chromosome X0 (*Saxetania paramonovi*) and with the neo-XY chromosomes at initial stages of chromosomal evolution (*Tropidauchen* species) in Iranian fauna of Pamphaginae grasshoppers allow us to suggest that translocation between an autosome and the original X chromosome in the karyotype evolution in this subfamily originated in the Iranian highlands.

## References

[B1] Alicata P Messina A Oliveri S (1976) Frequenza e distribuzione dei chiasmi in *Pamphagus marmoratus* Burm., *Acinipe calabra* (Costa) e *Ocneridia canonica* (Fish.) (OrthopteraPamphagidae). Animalia 3: 171–193.

[B2] Bey-Bienko GYa Mistshenko LL (1951) Locusts and Grasshoppers. Part 1. Fauna of the USSR and Adjacent Countries, Vol. 38, 400 pp. [In Russian]

[B3] Bolzán AD (2017) Interstitial telomeric sequences in vertebrate chromosomes: Origin, function, instability and evolution. Mutation Research 773: 51–65. 10.1016/j.mrrev.2017.04.002 28927537

[B4] Bugrov AG (1986) Neo-XY sex-chromosome determination in grasshoppers *Asiotmethis heptapotamicus heptapotamicus* (Zub) and *Atrichotmethis semenovi* (Zub) (OrthopteraPamphagidae). Tsitologiya 28: 117–119. [In Russian]

[B5] Bugrov AG (1996) Karyotypes of the short-horned Orthopteran insects (Orthoptera, Caelifera) from Russia, Kazakhstan, Central Asia, and the Caucasus. Folia Biologica (Krakow) 44(1–2): 15–25.

[B6] Bugrov AG Jetybayev IE Karagyan GH Rubtsov NB (2016) Sex chromosome diversity in Armenian toad grasshoppers (Orthoptera, Acridoidea, Pamphagidae). Comparative Cytogenetics 10: 45–59. 10.3897/CompCytogen.v10i1.6407 27186337 PMC4856925

[B7] Bugrov AG Warchałowska-Śliwa E (1997) Chromosome numbers and C-banding patterns in some Pamphagidae grasshoppers (Orthoptera, Acrididae) from the Caucasus, Central Asia, and Transbaikalia. Folia Biologica (Krakow) 45(3–4): 133–138.

[B8] Buleu OG Jetybayev IY Bugrov AG (2017) Comparative analysis of chromosomal localization of ribosomal and telomeric DNA markers in three species of Pyrgomorphidae grasshoppers. Comparative Cytogenetics 11(4): 601–611. 10.3897/compcytogen.v11i4.14066 29114356 PMC5672159

[B9] Buleu OG Jetybayev IY Chobanov DP Bugrov AG (2019) Comparative analysis of C-heterochromatin, ribosomal and telomeric DNA markers in chromosomes of Pamphagidae grasshoppers from Morocco. Comparative Cytogenetics 13(1): 61–74. 10.3897/CompCytogen.v13i1.32039 30854170 PMC6403196

[B10] Cabrero J Camacho JPM (1986) Cytogenetic Studies in Gomphocerine Grasshoppers. I. Comperative Analysis of Chromosome C-banding pattern. Heredity 56: 365–372. 10.1038/hdy.1986.58

[B11] Cabrero J Camacho JPM (2008) Location and expression of ribosomal RNA genes in grasshoppers: Abundance of silent and cryptic loci. Chromosome Research 16(4): 595–607. 10.1007/s10577-008-1214-x 18431681

[B12] Cabrero J Camacho JPM Pascual F (1985) Cytotaxonomic studies on pamphagids genus *Eumigus*. Detection of two chromosomal races in *E. monticola* (Rambur) (Insecta, Orthoptera). Caryologia 38(1): 1–12. 10.1080/00087114.1985.10797724

[B13] Camacho JPM Cabrero J Viseras E (1981) C-heterochromatin variation in the genus *Eumigus* (Orthoptera, Pamphagoidea). Genetica 56(3): 185–188. 10.1007/BF00057558

[B14] Fossey A (1985) Cytogenetic Research of the Short-Horned Orthoptera Insect from South Africa. Dr Sci. Dissertation, Pretoria University, Pretoria, 106 pp.

[B15] Fu P (1989) Karyotype C-banding staining on two species of the genus *Sinotmethis* B.-Bienko (Orthoptera, Pamphagidae). Hereditas (China) 11(3): 26–28.

[B16] Hewitt GM (1979) Grasshoppers and cricket. In: John B (Ed.) Animal Cytogenetics, 3. Insecta I. Orthoptera. Borntraeger, Berlin, Stuttgart, 170 pp 10.3897/compcytogen.v5i4.2307

[B17] Hodjat SH (2012) An update list of Pamphagidae Brumster 1840 (Insecta: Orthoptera) of Iran with a key to genera. Journal of Crop Protection 1(3): 261–270.

[B18] Jetybayev IE Bugrov AG Karamysheva TV Camacho JPM Rubtsov NB (2012) Chromosomal localization of ribosomal and telomeric DNA provides new insights on the evolution of Gomphocerinae grasshoppers. Cytogenetic and Genome Research 138(1): 36–45. 10.1159/000341571 22922814

[B19] Jetybayev IE Bugrov AG Ünal M Buleu OG Rubtsov NB (2017a) Molecular cytogenetic analysis reveals the existence of two independent neo-XY sex chromosome systems in Anatolian Pamphagidae grasshoppers. BMC Evolutionary Biology 17(1): 1–20. 10.1186/s12862-016-0868-9 28251879 PMC5333169

[B20] Jetybayev IY Bugrov AG Buleu OG Bogomolov AG Rubtsov NB (2017b) Origin and evolution of the neo-sex chromosomes in Pamphagidae grasshoppers through chromosome fusion and following heteromorphization. Genes 8(323). 10.3390/genes8110323 PMC5704236 29137168

[B21] King M John B (1980) Regularities and restrictions governing C-band variation in acridoid grasshoppers. Chromosoma (Berlin) 76(2): 123–150. 10.1007/BF00293413

[B22] Kuznetsova V Grozeva S Gokhman V (2019) Telomere structure in insects: A review. Journal of Zoological Systematics and Evolutionary Research 58: 127–158. 10.1111/jzs.12332

[B23] Mansueto C Vitturi R (1989) NORs location and C-banding pattern in spermatogenesis of Pamphagus ortolanii (Orthoptera, Acrididae). Caryologia 42: 303–311. 10.1080/00087114.1989.10796978

[B24] Massa B (2013) Pamphagidae (Orthoptera: Caelifera) of North Africa: key to genera and the an- notated check-list of species. Zootaxa 3700(3): 435–475. 10.11646/zootaxa.3700.3.7 26106736

[B25] Mirzayans (1998) Insects of Iran: the list of Orthoptera in insect collection of Plant pests & diseases Research Institute: Orthoptera (X), Pamphagidae (8) and Pyrgomorphidae (10). Tehran, 40 pp.

[B26] Mistshenko LL (1951) Revision of Orthoptera of the genus Tropidauchen Sauss. (Saltatoria – Orthoptera, Acrididae) and related genera. Dokladij Akademii Nauk USSR 57: 737–740. [In Russian] 14822858

[B27] Palacios-Gimenez OM Castillo ER Martí DA Cabral-de-Mello DC (2013) Tracking the evolution of sex chromosome systems in Melanoplinae grasshoppers through chromosomal mapping of repetitive DNA sequences. BMC Evolutionary Biology 13(1): 1–167. 10.1186/1471-2148-13-167 23937327 PMC3751140

[B28] Pinkel D Straume T Gray JW (1986) Cytogenetic analysis using quantitative, high-sensitivity, fluorescence hybridization. Proceedings of the National Academy of Sciences of the United States of America 83: 2934–2938. 10.1073/pnas.83.9.2934 3458254 PMC323421

[B29] Rubtsov N Karamysheva T Astakhova N Liehr T Claussen U Zhdanova N (2000) Zoo-FISH with region-specific paints for mink chromosome 5q: delineation of inter- and intra- chromosomal rearrangements in human, pig and fox. Cytogenetics and Cell Genetics 90: 268–270. 10.1159/000056786 11124531

[B30] Sahara K Marec F Traut W (1999) TTAGG telomeric repeats in chromosomes of some insects and other arthropods. Chromosome Research 7: 449–60. 10.1023/A:1009297729547 10560968

[B31] Santos JL Arana P Giraldez R (1983) Chromosome C-banding patterns in Spanish Acridoidea. Genetica 61: 65–74. 10.1007/BF00563233

[B32] Sergeev MG (1995) The general distribution of Orthoptera in the eastern parts of the Saharan-Gobian and Scythian Subregions. Acta Zoologica Cracoviensia 38(2): 213–256.

[B33] Shumakov EM (1963) Acridoidea of Afghanistan and Iran. Academie des Science de L’ URSS. 284 pp.

[B34] Ünal M (2016) Pamphagidae (Orthoptera: Acridoidea) from the Palaearctic Region: taxonomy, classification, keys to genera and a review of the tribe Nocarodeini I. Bolívar. Zootaxa 4206(1): 1–223. 10.11646/zootaxa.4206.1.1 27988545

[B35] Uvarov BP (1966) Grasshoppers and locusts. A handbook of general acridology (Vol. 1). London, Cambridge University Press, 481 pp.

[B36] Vitturi R Lannino A Mansueto C Mansueto V Stella M (2008) Silver-negative NORs in *Pamphagus ortolaniae* (Orthoptera: Pamphagidae). European Journal of Entomolology 105: 35–39. 10.14411/eje.2008.004

[B37] Vitturi R Mansueto C Ficarella P (1993) Heterochromatin variation in four species of the genus *Pamphagus* (Orthoptera: Pamphagidae) analyzed by C-banding. Biologisches Zentralblatt 112: 335–341.

[B38] Warchałowska-Śliwa E Maryańska-Nadachowska A Massa B (1994) Some new data on C-banding and NORs in three species of Pamphagidae (Orthoptera). Folia Biologica (Krakow) 42(1–2): 13–18.

[B39] Warchałowska-Śliwa E Grzywacz B Maryańska-Nadachowska A Heller K-G Hemp C (2020) Rapid chromosomal evolution in the bush-cricket *Gonatoxia helleri* Hemp, 2016 (Orthoptera, Phaneropterinae). Comparative Cytogenetics 14(3): 417–435. 10.3897/CompCytogen.v14i3.54422 32952902 PMC7473956

[B40] White MJD (1940) The origin and evolution of multiple sex-chromosome mechanisms. Journal of Genetics Springer 40: 303–36. 10.1007/BF02982496

[B41] White MJD (1973) Animal Cytology and Evolution. Cambridge, 961 pp.

